# Determination of Effective Factors on Survival of GI Cancers: Results of Five Years Follow up in Iranian Population

**DOI:** 10.5539/gjhs.v8n6p256

**Published:** 2015-11-17

**Authors:** Ali Zargar, Arash Miroliaee, Somayeh Ahmadi Gooraji, Aliakbar Hajaghamohammadi

**Affiliations:** 1 Department of Internal Medicine, Velayat Clinical Research Development Unit, Velayat Hospital, Qazvin University of Medical Sciences, Qazvin, Iran; 2 Velayat Clinical Research Development Unit, Velayat Hospital, Qazvin University of Medical Sciences, Qazvin, Iran

**Keywords:** survival, esophagus cancer, gastric cancer, colorectal cancer, cox regression

## Abstract

**Background:**

The gastrointestinal cancers are among the most common cause of cancer-related death and their long term survival is very low. This study was aimed to determine the effective factors on survival of gastrointestinal cancers among Iranian population during 5 years of follow up.

**Methods:**

In total, 157 patients diagnosed as gastrointestinal cancers from 2007 to 2009 in the only center of endoscopy in Alvand city, northwest of Qazvin province were included and followed for five years. The univariate and multivariate analysis were done using Kaplan-Meier method and the Cox model respectively.

**Results:**

Observations of 146 patients were analyzed (99 (67.8%) males and 47 (32.2%) females). The mean age was 64.73± 13.23 and 58.28±13.91 for females and males respectively. The one and three years survival rates for esophageal cancer were 28% and 9% and the one, three and five years survival rates for gastric cancer were 31%, 26% and 14% and for colorectal cancer were 96%, 86% and 75% respectively. In the univariate analysis, variables of age, educational level, ethnicity, smoking, type of cancer, stage of disease and type of treatment had significant effects on survival. In the multivariate analysis, the type of cancer and type of treatment affected the survival of patients as effective factors (p<005).

**Conclusion:**

Patients with esophageal cancer and those who underwent RT &/or CT are exposed to higher risk of death. Combination therapies (Surgery and adjuvant or neoadjuvant therapy) were related to be her survival. Early diagnosis and use of extended cancer screening programs seem necessary to improve survival.

## 1. Introduction

The GI Cancers are one of the most common cancers and cause of the cancer death in Asia and most developing countries such as Iran (Ghadimi, Mahmoodi, Mohammad, Zeraati, Rasouli, & Sheikhfathollahi, 2011; Pourhoseingholi, Fazeli, Ashtari, & FazeliBavand-Pour, 2013; Pourhoseingholi, Vahedi, & Baghestani, 2015). Generally, gastrointestinal malignancies are mainly occurring in stomach, esophagus and colorectal and are comprising more than 38% of all cancers and approximately accounting for more than half of the cancer mortality in Iran. Cancers of stomach, esophagus, and breast, prostate and colorectal after the skin cancer are the five common cancers in both sexes in Iran (Nasrabadi, Bahabadi, Hashemi, Valiee, & Seif, 2011).

Mortality rate of Gastrointestinal Cancers is more than the other cancers (Pourhoseingholi, Fazeli, Ashtari, & Fazeli Bavand-Pour, 2013). The esophageal cancer is the 8^th^ commonest cause of death globally with short survival rate of about 40% after one year of diagnosis. Total 5-year survival rate of esophageal cancer is estimated at 12% in Iran. Gastric cancer is the 4^th^ most common cause of death globally and its overall 5-year survival rate is less than 20% while colorectal cancer is the 5^th^ commonest oneand its incidence rate is stable or increasing in most countries. Overall 5-year survival rate of colorectal cancer was about 41% in Iran, and its proportion in people younger than 40 was more than those seen in western countries (R. Brown, & J. Ahnen, 2014; Yarhusseini, Sharifzadeh, Delpisheh, Veisani, Sayehmiri, & Sayehmiri, 2014; Dolatkhah, Somi, Jabbarpour Bonyadi, AsvadiKermani, Farassati, & Dastgiri, 2015).

The cause of gastrointestinal cancers is yet unknown and the role of genetic and environmental factors in their etiology vary according to each organ involved and also in their subtypes to the extent that the role of genetic factor is uncertain in esophageal cancer although its familial aggregations is reported from china (Chang-Claude et al., 1997; Li et al., 1989), Sweden and America (Hemminki & Jiang, 2002; Ji & Hemminki, 2006). In contrast the role of genetic factor is definite in FAP (familial adenomatous polyposis) contributing to less than 1% of colonic cancers to the extend that this autosomal dominant disease can be diagnosed in the carrier of this gene and cancer can be prevented by appropriate screening while non-carriers are safe (Spirio et al., 1993; Burt, DiSario, & Cannon-Albright, 1995). The MMR (mismatched DNA repair) gene is between of above two in such a way that this gene can be also found in 15% of sporadic colonic cancer (Jenkins et al., 2007) while there are cases of lynch syndrome contributing to 3 to 5% of colonic cancers that are devoid of this gene. If this gene is found in appropriate clinical setting its application is as same as that of APC (adenomatous polyposis coli) gene in FAP. In the subtype of hereditary diffuse gastric cancergermline mutations of the CDH1 gene inherited as an autosomal dominant patternif detected (Fitzgerald et al., 2010) has the same application as that of MMR as thismutation can also be found in sporadic gastric cancer (Humar et al., 2002). Role of genetic is also clear in the prognostication of colon cancer as detection of microsatellite instability indicates a more favorable outcome (Ogino et al., 2009). CEA (CarcinoEmbryogenic antigen) as a tumor marker is also used in colon cancer initially at diagnosis and for follow up to detect metastasis (Shakeri et al., 2013). Regarding the role of environmental factors, as for example the low oral hygiene (Locker et al., 2006) and N-nitroso compounds are well known in esophageal and gastric cancer (World Cancer Research Fund, 2007; Islami, Ren, Taylor, & Kamangar, 2009) to the extent that an esophageal belt cancer is found (Iran being part of it) and same is also true in gastric cancer to the extent that its endemic areas are known (Iran being part of it). Although the colon cancer was previously found to be more common in western countries because of their life style and high fat diet (Bener, 2011) but its prevalence is also rising in Middle East countries to the extent that nowadays its incidence in patients younger than 40 year is 15-35percentagainst 2-8percentseen in western countries (Dolatkhah, Somi, Bonyadi, Asvadi Kermani, Farassati, & Dastgiri, 2015). So far as the treatment is considered endoscopic treatment is used both in early stage (Technology status report evaluation, 2000) of these cancers and also as palliative in their advanced stages (Wu, Tsao, & Shyu, 2000). Surgery is mostly needed in them with or without chemo±radio therapy as neoadjuvant and adjuvant treatment or as the only treatment. Other cancers of this system are not reported in our cases because of being very few and therefore no discussion is made.

In the recent decades survival of patients with gastrointestinal cancer has increased by using surgical and nonsurgical methods but their overall survival has not changed. Generally as the gastrointestinal malignancies remain asymptomatic until they reach to an advanced stage therefore this delay in diagnosis results in their short survival (Samadi et al., 2007). In studies related to the survival rate of gastrointestinal malignancies different considerations including their demographics (age, sex, education, income and etc), social individual and therapeutic modalities, are mentioned to be as important measures affecting the survival rate of these patients. The aim of this study is to determine the effective factors on survival of patients with gastrointestinal cancers with respect to their demographic characteristics, type of cancer, type of treatment, stage of disease, location of tumor and morphology and is also to estimate their survival rate during a 5-years of follow up according to the type of gastrointestinal cancers.

## 2. Methods

This is a prospective study is conducted at Rahimian charity hospital (the only center of endoscopy) of Alvand city in Qazvin province. A total of 157 patients diagnosed as cancers of esophagus, stomach and colorectum based on the pathology report from 2007 to 2009 were included in this study and followed for five years. Survival status of patients was pursued by telephone contact with their relatives and also via the personal status registration organization of Qazvin province. Survival rate is calculated as per month. The effect of demographic variables including gender, age, income level, educational level, ethnicity (based on the ethnic groups studied in most studies in Iran), smoking, type of cancer, Location of tumor, morphology, stage of disease and also type of treatment including surgery only, CT and or RT (Chemotherapy/Radiotherapy) and combination therapy (Surgery and adjuvant or neoadjuvant therapy) was evaluated on the patients survival. The univariate analysis with Kaplan-Meier method and also the Cox model was used for the estimation of survival rate and determination of effective factors on it. Analysis of data was done at significant level of 0.05 by SPSS 16.

## 3. Results

In this study of a total of 157 patients suffering from gastrointestinal cancers, the survival observations of 146 patients was analyzed of whom99 were males (67.8%) and 47 were females (32.2%). The mean age of patients at the time of diagnosis was 64.73± 13.23 and 58.28±13.91 for females and males respectively. Out of the total of patients studied, 58.9% died during the study of whom 30.2% were females and 69.8% were males and 41% were still alive who were considered as of censored. The proportions of different type of gastrointestinal cancers were as 20% esophageal, 55% gastric and 24% colorectal.

**Figure 1:**
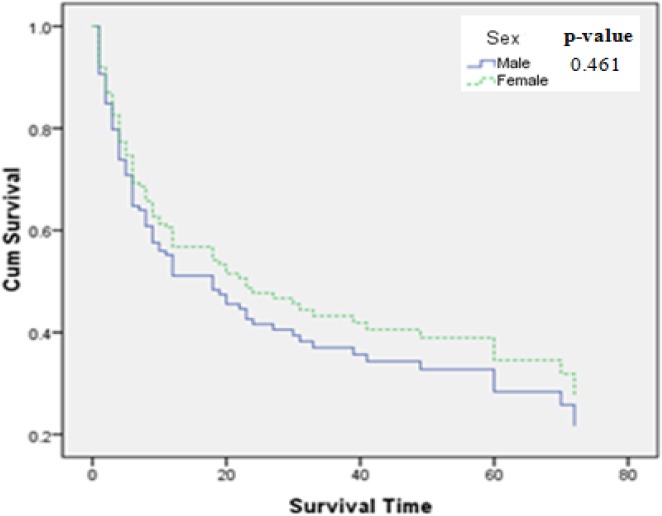
Kaplan-Meier curve of patients by sex

The median survival time was 18.7 months in total. The median survival time for esophageal, gastric and colorectal cancers was 9.32, 11.41 and 72 months respectively. The median survival time for esophageal cancer in term of sex; in females was 19.3 months and in males was 8 months; and for gastric cancer; in females was 12.88 months and in males was 10.97 months; and for colorectal cancer; in females was 72 months and in males was 74.20 months. Based on the results of life table, the one, three and five year survival rates were accordingly 45%, 36% and 27% in total. Besides, according to the type of gastrointestinal cancer, the one and three year survival rates were 28% and 9% for esophageal cancer and the one, three and five year survival rates were 31%, 26% and 14% for gastric cancer and 96%, 86% and 75% for colorectal cancer respectively. Demographic and cancer characteristics of patients with gastrointestinal cancers and their effects on survival rate were indicated in tables 1-3. Based on the univariate analysis using log-rank test, the variables of age, educational level, ethnicity, smoking, type of cancer, stage of disease and type of treatment had significant effects on survival. The significant results were not observed with the variables of sex (Table 1). Although this variable was not statistically significant but the median survival time was nearly more in females than males (Figure 1).

**Table 1:** Demographic characteristics of patients with gastrointestinal cancers and their effects on survival using log-rank test (univariate analysis)

Variable	Category	N (%)	p-value
Age group	< 62	66(45.2)	0.011*
	≥ 62	80(54.8)	

Sex	Male	99(67.8)	0.461
	Female	47(32.2)	

Income level	weak	60(46.2)	0.838
	Moderate	16(12.3)	
	Well	54(41.5)	

Educational level	illiterate	82(56.2)	0.002*
	Primary(school)	35(24)	
	Secondary(high school)	11(7.5)	
	University	13(8.9)	

Ethnicity	Fars	47(32.2)	0.024*
	Tourk	72(49.3)	
	Kord	5(3.4)	
	Lour	3(2.1)	
	Gilak	10(6.8)	

Smoking	Non-smoker	89(61)	0.011*
	Smoker	34(23.3)	

* Significant at level of 0.05.

**Table 2:** Cancer characteristics and their effects on survival using log-rank test (univariate analysis)

Variable	Category	N(%)	p-value
Type of cancer	Esophagus	30(20.5)	0.001*
	Gastric	81(55.5)	
	Colorectal	35(24)	

Type of treatment	Surgery	48(32.9)	0.006*
	RT &/or CT^a^	27(18.5)	
	Combination therapy^b^	59(40.4)	

* Significant at level of 0.05.

a. RT: radiotherapy, CT: chemotherapy.

b. Surgery and adjuvant or neoadjuvanttherapy.

**Table 3:** Cancer characteristics and their effects on survival rate by type of gastrointestinal cancer using log-rank test (univariate analysis)

variable	category	Esophageal cancer		Gastric cancer		Colorectal cancer	
		N (%)	P-value	N (%)	P-value	N (%)	P-value
Location of Tumor	Proximal	2(6.7)	0.941	28(34.6)	0.082	-	0.635
	Middle	12(40)		13(16)		-	
	Distal	16(53.3)		37(45.7)		-	
	Holestomach	-		3(3.7)		-	
	Colon	-		-		18(51.4)	
	Rectum	-		-		17(48.6)	
Morphology	Intestinal Ac^a^	5(16.7)	0.366	64(79)	0.478	30(85.7)	0.143
	Diffuse Ac	-		8(9.9)		-	
	Mucinproducing Ac	3(10)		9(11.1)		5(14.3)	
	KeratinizingScc^b^	17(56.7)		-		-	
	Non KeratinizingScc	5(16.7)		-		-	
Stage of disease	Local	13(43.3)	0.131	26(32.1)	0.0003	13(37.1)	0.292
	Regional	6(20)		33(40.7)		16(45.7)	
	Metastatic	11(36.7)		22(27.2)		6(17.1)	

a. Adenocarcinoma.

b. Squamous cell carcinoma.

Also, based on the univariate analysis on cancer characteristics, significant results wasnot observed with the variables of tumor location (in esophageal cancer including proximal/middle/distal; in gastric cancer including proximal/middle/distal/whole stomach; in colorectal cancer including colon/rectum) and morphology (in esophageal cancer including intestinal Adenocarcinoma(AC)/Mucin producing AC/keratinizing Squamous cell carcinoma (SCC)/not keratinizing SCC; in gastric cancer including intestinal AC/diffuse AC/Mucin producing AC; in colorectal cancer including intestinal AC/Mucin producing AC). In case of the stage of disease, significant survival difference was only observed for gastric cancerthat is probability due to a large sample size for gastric cancer (p<005); for this reason, this variable did not consider in multivariate analysis (Tables 2 and 3).

**Figure 2:**
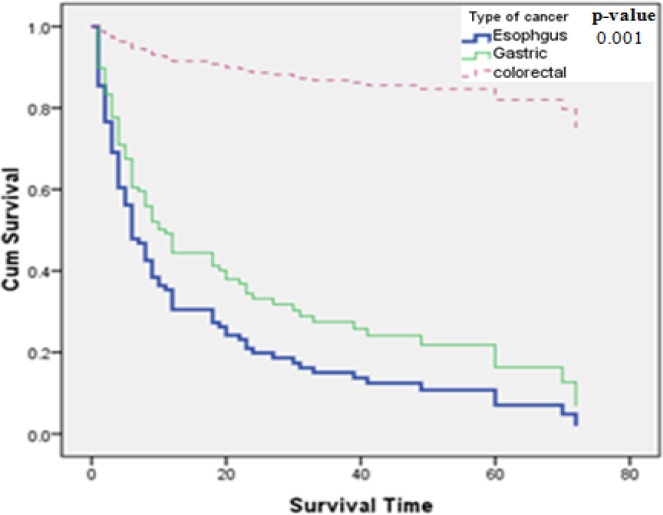
Kaplan-Meier curve of patients by type of gastrointestinal cancer.

Besides, the multivariate analysis using forward Cox regression model was made after assessing the assumption of constant relative hazard during the time by using log-log plot. Based on the results of Table 4, the variables of cancer type and type of treatment were considered to be effective significant factors on survival of patients. The hazard ratio(HR) for esophageal and gastric cancers were about 12.39 and 11.65 times more than that seen in colorectal cancer accordingly (p<005). In fact, patients with esophageal cancer are exposed at a higher risk of death. The survival curve of each gastrointestinal cancer is given in figure (2) separately.

**Table 4:** Results of fitting the cox regression model (multivariate analysis)

Variable	category	coefficient	Standard Error	p-value	HR^*^
Type of cancer	Esophagus	2.52	0.649	0.001**	12.39
	Gastric	2.46	0.607	0.001**	11.65
	Colorectal	-	-	-	Reference

Type of treatment	Surgery	0.175	0.334	0.600	1.192
	RT &/or CT^a^	0.739	0.317	0.020**	2.094
	Combination therapy^b^	-	-	-	Reference

* Hazard Ratio.

** Significant at level of 0.05.

a. RT: radiotherapy, CT: chemotherapy.

b. Surgery and adjuvant or neoadjuvanttherapy.

**Figure 3:**
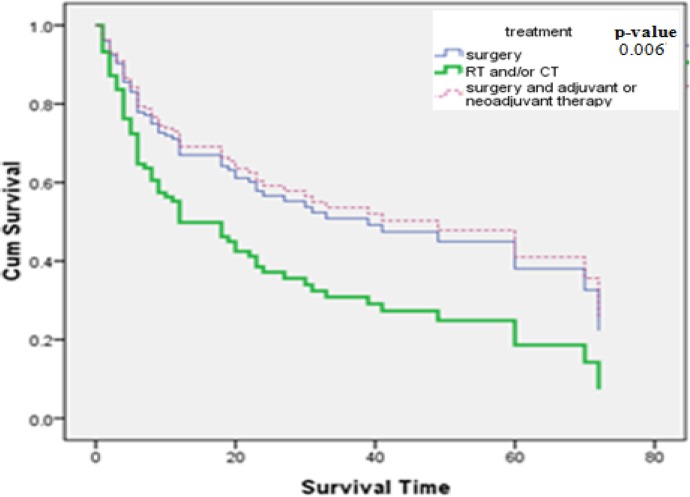
Kaplan-Meier curve of patients by type of treatment.

Also, the hazard ratio in patients underwent RT &/or CT were 2 folds greater than that seen in patients under combination therapy (p<005) but the hazard ratio in patients who underwent surgery only was 19% more than those with combination therapy (p<005). In other word, RT &/or CT showed an increased risk of death as compared to surgery only or those with combination therapy (Surgery and adjuvant or neoadjuvant therapy) (Figure 3).

## 4. Discussion

This study was aimed to determine the effective factors on survival of gastrointestinal cancers among Iranian population during 5 years of follow up. Although in most of the recent studies survival of upper and lower gastrointestinal cancer is investigated separately but the aim of this study is to determine the effective factors on survival of gastrointestinal cancers combined. In our study based on the results of life table; the survival rates for gastric cancer were more or close to the survival rates of other studies with overall 5-years survival of 16.2% (Bashash, Hislop, Shah, Le, Brooks-Wilson, & Bajdik, 2008) and 18% (Mingol et al., 2015) and 1,3,5 years survival rates of 41%, 13%, 5.4% respectively (Veisani, Delpisheh, Sayehmiri, & Rahimi, 2013). In our study, the survival rates for colorectal cancer were approximately higher than those reported in most recent studies with 1,3,5 years survival rates of 84%, 54%, 41% (Moradi, Khayamzadeh, Guya, Mirzaei, Salmanian, Rakhsha, & Akbari, 2009), and survival rates of 72%, 54%, 47% (Mehrkhani, Nasiri, Donboli, Meysamie, & Hedayat, 2009), and overall 5-years survival of 38.6% (Laohavinij, Maneechavakajorn, & Techatanol, 2010). This difference in survival of colorectal cancer in our study is perhaps due to an earlier diagnosis in our open access endoscopy center and is also because of improvement in their treatment due to the advance in therapeutic protocols including the neoadjuvant one.

In our study, the one and three years survival rates for esophageal cancer were lower than those reported with 55%, 18% in a study by Mirinezhad et al., (2012), and as 23%, 15% in a study by Ghadimi et al., (2012), and as 51.3%, 20.1% in a study by Kandaz et al., (2012), and as 49%, 24% in a study by Veisani et al., (2013). This difference may be due to the low sample size of esophageal cancer patients in our study.

The results of present study based on univariate analysis using log rank test showed that, the variables of age, educational level, ethnicity, smoking, type of cancer and type of treatment had a significant survival differences but sex, location of tumor and morphology has not been indicated significant in survival differences. Although in our study the variable of sex did not have a significant effect on survival but the survival rate of females has been more than of males. Some Studies confirm the result of our study (Ghadimi, Mahmoodi, Mohammad, Rasouli, Zeraati, & Fotouhi, 2012; Bohanes et al., 2012; Majek et al., 2013; Veisani, Delpisheh, Sayehmiri, & Rahimi, 2013). Besides, in the evaluation of survival of patients according to the type of gastrointestinal cancers, our result showed that in the cancers of esophagus and stomach, females have had a better survival; although the survival rate between the both sexes have been almost alike in colorectal cancer. In most of studies, females had better survival in total (Bashash, Hislop, Shah, Le, Brooks-Wilson, & Bajdik, 2008, 2011; Gaur, Sepesi, Hofstetter, Correa, Bhutani, Watson, & Swisher, 2011). In most of studies a significant relation has been found between variable of age and survival rate as patients younger than 65 years had a better survival (Mehrkhani, Nasiri, Donboli, Meysamie, & Hedayat, 2009; Yang et al., 2011). In another study, the covariate of age had inverse relation with survival of patients with esophageal cancer (Shitara et al., 2010). In our study, patients younger than 62 years suffering from gastrointestinal cancers (esophagus, stomach and colorectal cancer) had longer survival. In our study based on the univariate analysis, university degree, non-smoking and receiving surgery and combination therapies implied on better survival. In a study, the variable of smoking had inverse effect on survival of patients with upper gastrointestinal cancer (Sundelöf, Lagergren, & Ye, 2008) which was similar to our findings.

In the concomitant study of variables, based on the results of multivariate analysis using Cox model, variables of type of cancer and type of treatment had statistically significant effect on the patients’ survival which is also mentioned as the effective factors on survival in univeriate analysis in present study.

Our results showed that patients with esophageal cancer were exposed at a highest risk of death. In a study on survival of patients with upper gastrointestinal cancers among Iran and in British Columbia (multi-ethnic population), Canada, result of multivariate analysis has proposed that the survival of patients suffering from gastric cancer has been better than that of esophageal cancer (Bashash, Shah, Hislop, Brooks-Wilson, Le, & Bajdik, 2011); while in another study in Iran, patients with esophageal cancer had a slightly better survival which could have been due to a wider Screening program of this cancer in north-west of this country (samadi et al., 2007). Besides, in a study of patients’ survival with and without metastatic stage, it is reported that patients with colorectal cancer have a longer life than patients with gastric cancer (Moghimi-Dehkordi, Safaee, & Zali, 2009). These results confirm our findings which are briefly indicated in Table 5.

**Table 5:** Results of other studies by type of gastrointestinal cancers

study	Type of gastrointestinal cancer	%	Mean age	Male (%)	Female (%)	Median survival time(month)	HR	Effective factors on survival using Cox model
Moghimi-Dehkordi et al. in 2009	Gastric cancer	60	55.9	61	29	42.46	2.25	Distant metastasis, grade of tumor
	Colorectal cancer	39.8		71	38.8	104.9	1.92	
Samadi et al. in 2007	Gastric cancer	66.4	64	77.8	22.2	11.8	Reference	Type of treatment, smoking status
	Esophageal cancer	33.5		53.9	46.1	12.1	0.86	
Bashash et al. in 2011	Gastric cancer	20	67	78.9	21.1	20	0.64	Ethnicity, type of treatment, tumor location and histology, sex
	Esophageal cancer	60		71.4	28.6	7	1.13	

The choice of treatment type (with or without surgery) has solely been based on staging and probability of response to treatment and informed consent of patients. In another result of multivariate analysis in present study, patients who underwent RT &/or CT were exposed at a higher risk of death, which may be related to a higher stage of their disease due to a delayed diagnosis. In study on patients suffering from upper gastrointestinal cancers, results of multivariate analysis showed that patients who underwent surgery had 51% lower mortality than those who received non-surgical treatment (samadi et al., 2007). In a study by Yang et al., (2011) using Cox model, gastric cancer patients who underwent surgery (HR=0.6) had better survival than those who underwent radiotherapy (HR=0.8). In a study by shirata et al., (2010) using Cox model, esophageal cancer patients who underwent surgery (HR=0.81) had better survival than those who underwent chemo radiotherapy (HR=1.48). In another study on upper gastrointestinal cancers, results of univariate analysis showed that esophageal cancer patients who received surgery had more survival than those who received chemotherapy or radiotherapy (median survival time of 12 v.s 5), also gastric cancer patients who received surgery had more survival than those who received chemotherapy or radiotherapy (median survival time of 12 v.s 9 and 7 respectively) (Veisani, Delpisheh, Sayehmiri, & Rahimi, 2013). In a study on esophageal cancer in Iran, patients who received chemotherapy or chemo radiotherapy along with Surgery (HR=0.19) had better survival than patients who received these treatments without Surgery such as only chemotherapy (HR= 0.79) or only radiotherapy (HR=0.63) (Aghcheli et al., 2011). In another study, esophageal cancer patients with only surgical treatment were almost at higher risk of death than those who did not have surgery (HR= 1.14) (Mirinezhad, Somi, Jangjoo, Seyednezhad, Dastgiri, Mohammadzadeh, Naseri, & Nasiri, 2012). Based on other results, surgery and/or combination therapy such as chemo radiotherapy (Kandaz, Ertekin, & Bilici, 2012) followed by surgery (Siersema & van Hillegersberg, 2008) or complete surgical resection and adjuvant chemotherapy (Laohavinij, Maneechavakajorn, & Techatanol, 2010) are Related to decrease the risk of death in gastrointestinal cancer. Results of these studies confirm our findings.

## 5. Conclusion

This study has attempted to determine the effective factors on survival of patients with gastrointestinal cancer. Results of this study indicate that the type of cancer and type of treatment are considered as potential risk factors on survival of patients. In the other word, patients with esophageal cancer undergoing RT &/or CT are exposed to a higher risk of death. Our findings also indicate that surgery and/or combination therapy are associated with the better survival in patients with gastrointestinal cancer. Definitely, the best therapeutic response for patients is surgical operation, and in practice also surgical operation with acceptance of its higher risk is probably a practical recommendation. Therefore our findings can provide beneficial information to extend the cancer screening programs in order to achieve an earlier diagnosis to reduce mortality and its related risk factors.
